# Modulation of the H^+^/ATP coupling ratio by ADP and ATP as a possible regulatory feature in the F-type ATP synthases

**DOI:** 10.3389/fmolb.2022.1023031

**Published:** 2022-10-05

**Authors:** Paola Turina

**Affiliations:** Department of Pharmacy and Biotechnology, University of Bologna, Bologna, Italy

**Keywords:** ATP synthase, H^+^/ATP, coupling ratio, unisite catalysis, ATP-Pi exchange, sulfite, 17*β*-estradiol, energy homeostasis

## Abstract

F-type ATP synthases are transmembrane enzymes, which play a central role in the metabolism of all aerobic and photosynthetic cells and organisms, being the major source of their ATP synthesis. Catalysis occurs *via* a rotary mechanism, in which the free energy of a transmembrane electrochemical ion gradient is converted into the free energy of ATP phosphorylation from ADP and Pi, and vice versa. An ADP, tightly bound to one of the three catalytic sites on the stator head, is associated with catalysis inhibition, which is relieved by the transmembrane proton gradient and by ATP. By preventing wasteful ATP hydrolysis in times of low osmotic energy and low ATP/ADP ratio, such inhibition constitutes a classical regulatory feedback effect, likely to be an integral component of *in vivo* regulation. The present miniview focuses on an additional putative regulatory phenomenon, which has drawn so far little attention, consisting in a substrate-induced tuning of the H^+^/ATP coupling ratio during catalysis, which might represent an additional key to energy homeostasis in the cell. Experimental pieces of evidence in support of such a phenomenon are reviewed.

## 1 Introduction

F-type ATP synthases (“ATP synthases” in the following) are highly conserved transmembrane enzymes, found in most eubacteria, mitochondria and chloroplasts (Boyer, 1997; Kinosita et al., 1998; Leslie and Walker, 2000; Junge and Nelson, 2015; Kühlbrandt, 2019), where they represent the main source of ADP phosphorylation. They convert the free energy of the transmembrane electrochemical H^+^-gradient (“H^+^-gradient” in the following)—or Na^+^-gradient, in some organisms (Dimroth, 1990)—into the free energy of ATP phosphorylation from ADP and Pi, and vice versa, as first proposed by the chemiosmotic theory (Mitchell, 1961). They do so by a rotary mechanism, in which, viewed from the hydrophobic transmembrane F_O_ sector to the hydrophilic F_1_ head, the rotor moves either clockwise (synthesis), or anti-clockwise (hydrolysis) (Diez et al., 2004; Iino and Noji, 2006; Noji and Ueno, 2022).

**FIGURE 1:**
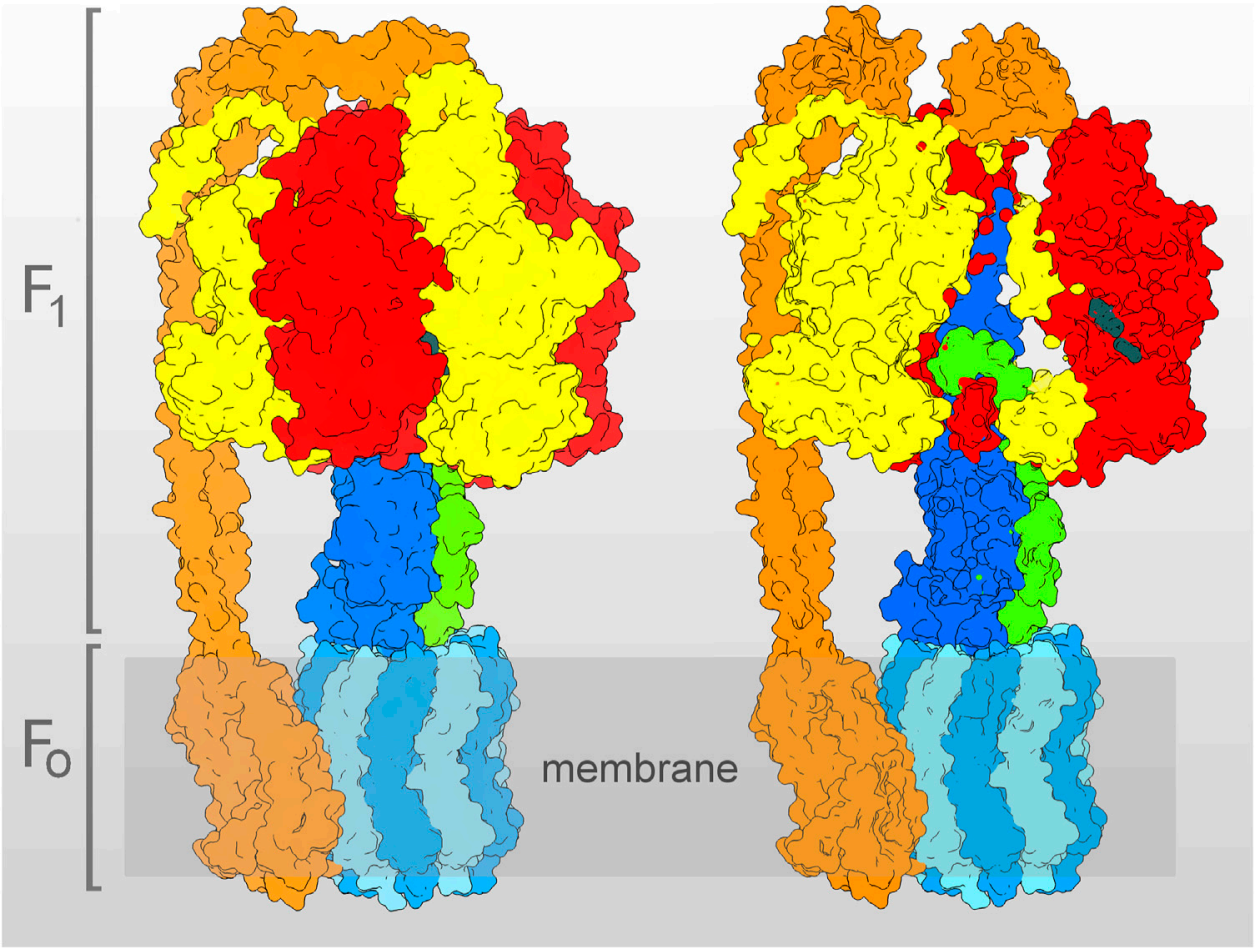
Molecular model of the *E. coli* ATP synthase, determined by cryo-EM (Sobti et al., 2020, PDB entry 6oqr, with the complex in the ε-subunit “up” state–see Section 3). On the left, the whole model is shown, on the right, the same model is clipped, to show the *γ*- (blue) and *ε*- (green) subunits filling the inner cavity of the *α*_3_ *β*_3_-barrel (*α*-subunit in yellow, *β*-subunit in red). The c_10_-oligomer (light blue), together with the *γ*- and *ε*-subunits, build the rotor, while the stator is constituted by the α_3_β_3_-barrel and by the peripheral stalk (orange), composed by the *δ*-, b_2_-, and a-subunits. In the clipped model, the ADP and Pi bound to the catalytic site of the *β*_1_ are visible (dark gray). The model was represented using USCF Chimera (Pettersen et al., 2004).

F_1_ contains a ring of three alternating *α*- and *β*-subunits, the latter bearing the three catalytic sites, at which ATP synthesis/hydrolysis occurs (Figure 1). The H^+^ translocate within F_O_, at the interface between the hydrophobic stator and rotor parts. The hydrophobic rotor part is a ring-shaped oligomer of c-subunits, which, during synthesis, transmits the torque to the hydrophilic rotor subunits *γ* and *ε*. The latters in turn rotate within the central cavity of the *α*_3_ *β*_3_-barrel, thus inducing the cyclic conformational changes in the *β*-subunits, which elicit substrate binding and product release. The number of c-subunits varies in different organisms, from 8 in the bovine mitochondrial enzyme, to 17 in the human pathogen *Burkholderia pseudomallei* (Kühlbrandt, 2019).

The rotary mechanism of F-type ATP synthases is shared by the related type-A ATP synthases (Archaebacteria) and type-V ATPases (vacuolar and other cellular membranes), the latter functioning *in vivo* for ATP hydrolysis (Zubareva et al., 2020). The three enzyme types also share some regulatory features, in particular the non-competitive inhibition by low ADP concentrations, which locks the enzyme in an inhibited conformation (Vasilyeva and Forgac, 1998; Feniouk and Yoshida, 2008; Sielaff et al., 2018; Zubareva et al., 2020). In the F-type enzymes, the tightly bound ADP is released, thus recovering the catalytic activity, at high ATP concentrations or high H^+^-gradients (Lapashina and Feniouk, 2018), by switching to a different conformation, which is metastable in the absence of a H^+^-gradient (Fischer et al., 2000, and references therein). By preventing the thermodynamically favored ATP hydrolysis at low ATP/ADP ratio and low H^+^-gradient, such regulation appears to be functional for cellular energy conservation in times of energy depletion.

Since every c-subunit bears one H^+^ binding site, and the *α*_3_ *β*_3_-barrel bears three catalytic sites, the rotary mechanism implies that the maximum possible H^+^/ATP stoichiometry is given by the number of c-subunit divided by three (Läuger, 1984; Junge, 1999; Watt et al., 2010). Bulk measurements in the chloroplast and mitochondrial enzymes, near to the thermodynamic equilibrium between the free energies of H^+^-gradient and ADP phosphorylation, have yielded values of the effective number of H^+^ translocated for each ATP molecule hydrolyzed/synthesized—the “H^+^/ATP coupling ratio” (Läuger, 1984)—close to, but 10%–15% smaller than, their respective c/*β* ratios (Van Walraven et al., 1996; Petersen et al., 2012, but see Soga et al., 2017, for even closer values in *Bacillus* PS3). Such less-than-c/*β* values are eventually consistent with some internal energy dissipation within the molecular motor (Martin et al., 2014; Hou and Wang, 2021), including, e.g., the H^+^-gradient energy used for maintaining otherwise metastable enzyme states (Turina et al., 2016), or the ATP binding/hydrolysis energy used for other “priming” reactions (see Section 3).

Since the rotary mechanism of the ATP synthase has been experimentally shown (Noji et al., 1997), much attention has been devoted to investigating, in single ATP synthase molecule experiments, the remarkably high tightness featured by its chemomechanical coupling (Kinosita et al., 2000, 2004; Toyabe et al., 2011; Zimmermann and Seifert, 2012; Saita et al., 2015). While such high efficiency in chemomechanical coupling can indeed be considered an evolutionary optimized feature of this “splendid molecular machine” (Boyer, 1997), on the other hand, cellular systems have also been shown to harbor sophisticated devices for fine-tuning the overall energy conversion efficiency. The mitochondria themselves are a prime example of regulated H^+^-leak mechanisms, with the Uncoupling Protein 1 and the ADP/ATP carrier considered to play the main role in the regulation of thermogenesis and reactive oxygen species production (Cadenas, 2018; Demine et al., 2019; Bertholet and Kirichok, 2022), or with their permeability transition pore deciding between cell life and death (Bernardi et al., 2021). The close cousins V-type ATPases have been shown to work with variable coupling ratios, proposed to be functional to variable acidification needs (Davies et al., 1994; Müller and Taiz, 2002; Kettner et al., 2003; Forgac, 2007; Saroussi and Nelson, 2009). In addition, variable coupling ratios in the ATP synthase, according to energy conditions, have been reported in whole bacterial cells (Gober and Kashket, 1984; Vink et al., 1984; Cook and Russell, 1994). Possibly, the marvel at the high tightness of energy conversion within the ATP synthase has let researchers in the field assume that its evolution, at a given c/*β* stoichiometry, had only taken place towards the highest possible coupling ratio. To my knowledge, very little work has been devoted so far to investigating the possibility that such coupling ratio could itself be subjected to regulation.

Purpose of the present work is to propose the existence of a built-in mechanism able to modulate the H^+^/ATP coupling ratio within the F-type ATP synthases, eventually useful for *in vivo* energy homeostasis, by highlighting some experimental evidence which is consistent with such hypothesis.

## 2 Nucleotide- and ligand-induced changes in the H^+^/ATP coupling ratio

### 2.1 Nucleotides

A change in coupling ratio, dependent on the ADP concentration, has been reported and investigated in bacterial ATP synthases (Turina et al., 2004; D’Alessandro et al., 2008). Measurements were carried out in the hydrolysis direction, progressively reducing the ADP concentration by an ADP trap at constant ATP concentration, thereby observing a corresponding reduction in the number of H^+^ pumped per hydrolyzed ATP. In both tested bacterial species, the less-coupled hydrolysis was inhibited by Fo inhibitors, oligomycin in *Rb. capsulatus,* DCCD in *E. coli*. The data could be most easily explained by the ATP synthase existing in, at least, two interconvertible states, differing by their own coupling ratios, with the higher coupling-ratio state (E_C_) favored by ADP binding, and the lower coupling-ratio state (E_U_) favored by ATP binding. ATP and ADP would compete for the same site, the occupancy of which, with either nucleotide, would determine the switch between the two conformations. That site could thus constitute a direct regulatory “sensor” of the cellular ATP/ADP ratio.

In D’Alessandro et al. (2011), D’Alessandro et al. (2017) two ADP binding sites were identified by their different functional effects and different apparent K_d_s, both in the sub-micromolar range, the tighter site being associated with the change in coupling ratio, and the other one with inhibition of ATP hydrolysis. Given the high-affinity binding of ADP to one of the three catalytic sites on *E. coli* F_1_ (Cingolani and Duncan, 2011), a possible scenario is that the two ADP binding sites can be identified as the same catalytic site, showing different properties in the two different enzyme conformations (E_U_ and E_C_).

In a nutshell, the above scenario implies that E_U_ is stabilized by ATP and that E_C_ is stabilized by ADP. We know that the tightly bound ADP fully inhibits catalysis, and that an H^+^-gradient is needed to release the tightly bound ADP and let the (fully coupled) rotary catalysis take place for several turnovers. Most likely, also the E_U_ needs some triggering event to get rid of the bound ATP, to be able to engage in its less coupled, but still rotary, catalysis. ATP binding/hydrolysis to another catalytic site could be such an event.

Previously, measurements of ATP hydrolysis in parallel with H^+^ pumping by (Shigalowa et al., 1985; Strotmann et al., 1986) had also supported the existence of an uncoupled form of the ATP synthase. According to the authors, the data could be best explained by postulating that a non-hydrolyzed ATP, bound to the ATP synthase in place of a tightly bound ADP, would induce a partially uncoupled ATP hydrolysis.

Interestingly, high concentrations of ATP were reported to induce a partial uncoupling of the V-ATPase pump (Arai et al., 1989; Shao and Forgac, 2004).

### 2.2 Sulfite

The most evident effect of sulfite on the ATP synthase is a strong increase of the hydrolysis activity, which has been associated with the release of the inhibitory, tightly bound ADP (Vasilyeva et al., 1982; Larson et al., 1989; Murataliev and Boyer, 1992). While the presence of ATP could slowly release the tightly bound ADP during catalysis, the presence of sulfite significantly accelerated the ATP-dependent reactivation of the ADP-inhibited complex. However, sulfite was shown to inhibit ATP synthesis, in a way not readily explained by competition with Pi (Pacheco-Moisés et al., 2002) and, consistently with the latter inhibition, was also shown to decrease the H^+^/ATP coupling ratio, as measured in the hydrolysis direction, both in the V-type ATPase (Kibak et al., 1993), and in the ATP synthase (Cappellini et al., 1997). The sulfite-activated hydrolysis was still completely inhibited by F_O_ inhibitors, such as oligomycin, venturicidin, DCCD (Moyle and Mitchell, 1975; Zhang et al., 1993; Cappellini et al., 1997; Pacheco-Moisés et al., 2000; Pacheco-Moisés et al., 2002).

The effects of sulfite can be interpreted in light of the scenario put forward in Section 2.1. The contiguity of ADP and sulfite in the tight ADP-binding site can be envisaged to mimic the structure of an ATP, and therefore to trigger the E_C_ → E_U_ conversion in the ADP-inhibited ATP synthase molecules. Such sulfite-induced conversion would cause the observed decrease in the H^+^/ATP ratio of hydrolysis, and the lower synthesis rate at constant H^+^-gradient. The proposal by Moyle and Mitchell (1975) and Pacheco-Moisés et al. (2000), that the sulfite-induced active state is associated with structural modifications of the enzyme, supports the above interpretation.

Other oxyanions, structurally similar to sulfite, have been less investigated (Walker, 1994). Selenite is known to increase hydrolysis (see, e.g., Jarman et al., 2021), and a systematic investigation might show that it is uncoupling as well. Carbonate has long been known to be activating for hydrolysis, and more recently it has also been reported to inhibit synthesis in submitochondrial particles (Lodeyro et al., 2001). The structurally close chloroform was also reported to change the H^+^/ATP ratio (Rottenberg, 1983).

### 2.3 ATP-Pi exchange

The ATP-Pi exchange is due to the ATP synthesis catalyzed by the ATP synthase in the presence of Pi and of a high ATP/ADP ratio, which induces an ATP-generated H^+^-gradient. It is called that way since the newly formed ATP is detected as the radioactive ATP generated when the only radioactive reagent at the start of the reaction was Pi. Lodeyro et al. (2001) excluded the possibility of an uncoupling effect of carbonate, based on their finding that it activated the ATP-Pi exchange. An alternative interpretation can be proposed on the basis of the E_U_ ↔ E_C_ hypothesis, since the carbonate-induced higher E_U_/E_C_ ratio would decrease the rates under the experimental conditions normally used for measuring ATP synthesis (low ATP/ADP ratio), but could increase the H^+^-gradient, and thus the ATP synthesis rates, during ATP-Pi exchange (high ATP/ADP ratio, see below). Notice that sulfite has also been shown to increase the rate of ATP-Pi exchange (Pacheco-Moisés et al., 2002).

Even though a high ATP/ADP ratio is a most frequent condition in the cell, the experimentally observed ATP-Pi exchange still represents an energetic conundrum for an ATP synthase with a homogeneous H^+^/ATP ratio, since substantial ATP synthesis is found under nearly prohibitive conditions for such reaction (very high ATP/ADP ratio and low H^+^-gradient). However, the co-occurrence of E_C_ and E_U_ during the ATP-Pi exchange could represent an easy solution for that enigma, since E_U_, if its lower H^+^/ATP coupling ratio was due to a lower H^+^/ATP stoichiometry (Section 4), would represent the steeper H^+^-gradient producer, and E_C_ the better H^+^-gradient consumer, thus allowing substantial synthesis rates by concomitant maximal measurable hydrolysis rates. The co-occurrence of two different active conformations (E_C_ and E_U_) would also agree with the results obtained by (Shoshan and Shavit, 1979), who showed that Fab fragments against F_1_ inhibited ATP-Pi exchange, but not light-driven synthesis, implying that an additional ATPase conformation (E_U_) played a major role during ATP-Pi exchange, different from the one (E_C_) catalyzing the light-driven synthesis.

### 2.4 Ca^2+^

The ATP synthesis/hydrolysis, catalyzed by the ATP synthase, requires Mg^2+^. When Mg^2+^ is substituted by Ca^2+^, the synthesis is inhibited, while the hydrolysis remains high, and still sensitive to the F_O_-inhibitors DCCD, oligomycin and venturicidin, but it becomes uncoupled from H^+^-translocation, as reported by several groups (e.g., Pick and Weiss, 1988; Strid and Nyrén, 1989; Casadio and Melandri, 1996; Papageorgiou et al., 1998). Uncoupling by Ca^2+^ has also been reported in V-type ATPases (Crider and Xie, 2003). The hydrolysis of CaATP was later shown capable of sustaining the rotary motions of the *γ*-subunit in single molecule detection, with a rate comparable to that induced by MgATP (Tucker et al., 2004). More recently, in purified and reconstituted bovine ATP synthase, Urbani et al. (2019) showed that Ca^2+^ causes channel-like dissipation of the ATP hydrolysis-induced H^+^-gradient, and does so even more in the presence of activators of the permeability transition pore.

Similar as proposed for sulfite (Section 2.2), the underlying reasons for Ca^2+^-uncoupling might be related to the ADP/ATP occupancy of the high-affinity catalytic site. Both by direct binding experiments (Maldonado et al., 1998) and by measuring the extent of water oxygen incorporation into released ATP (Kohlbrenner and Boyer, 1983), the same conclusion was reached, that Ca^2+^ in place of Mg^2+^ strongly accelerated ADP release from the high-affinity catalytic site on the enzyme. The E_C_ ↔ E_U_ interconversion would then be significantly shifted by Ca^2+^ toward the less coupled E_U_.

### 2.5 Other ligands

The human hormon 17*β*-Estradiol (E2) was found to directly bind to a subunit of the mitochondrial ATP synthase (Zheng and Ramirez, 1999) and to inhibit ATP synthesis (Massart et al., 2002). More recently, in simultaneous measurements of H^+^-gradient and ATP synthesis, Moreno et al. (2013) have reported that E2 decreased the H^+^/ATP ratio in the synthesis direction, and that such an effect was dependent on the presence of non-hydrolyzed ATP. As proposed above (Sections 2.1–2.4), such decrease could be due to a shift of the E_C_ ↔ E_U_ equilibrium toward the less coupled E_U_, induced by E2 binding to the enzyme.

Interestingly, the mitochondrial ATP synthase has also been reported to be a target for several phytoestrogens, such as quercetin, resveratrol, curcumin (Zheng and Ramirez, 2000), for the anorexigenic peptides enterostatin (Berger et al., 2002; Lindqvist et al., 2008) and Angt_Human [448–462] (Sasaki et al., 2022), as well as for the anti-apoptotic peptide Bcl-xL, whose binding to the *β*-subunit was reported to decrease an ATP-dependent ion leak within the ATP synthase (Alavian et al., 2011).

The phytotoxin tentoxin showed also an interesting behavior, reminiscent of that of sulfite. At low concentrations, it was reported to increase the affinity of the non-hydrolyzable ATP analog AMPPNP for the high-affinity nucleotide-binding site (Selman and Selman-Reimer, 1979), thus blocking the enzyme, but then favoring the E_U_ conformation at higher concentrations. Consistently, higher tentoxin concentrations reactivated hydrolysis and rotation (Meiss et al., 2008), and at a lower H^+^/ATP ratio (Sigalat et al., 1995).

## 3 Nucleotide-induced changes in the ATP synthase conformation under unisite conditions

When the ratio between substrates and ATP synthase is less than one, catalysis has been shown to occur at the high-affinity site, both in synthesis and hydrolysis direction—the so-called “unisite” catalysis, which is several orders of magnitude slower than multisite steady-state catalysis (Penefsky and Cross, 1991). Though unisite catalysis has often been considered a step cooperatively integrated to each turnover of the multisite catalysis, it has also been shown to take place in the absence of rotation (García and Capaldi, 1998), a result in agreement with those of Bullough et al. (1987), and with a recent cryo-EM study, which indicates that unisite catalysis is an initial reaction that is distinguished from steady-state rotary catalysis (Nakano et al., 2022). In addition, Sakaki et al. (2005) measured the same rotation rates from mM down to nM ATP concentrations, without detecting any transition from multisite to unisite rates.

In (Turina and Capaldi, 1994), the kinetics of unisite catalysis was found to match the kinetics of the emission changes of a fluorescent probe, attached to a site-directed cysteine in the *γ*-subunit as a reporter of conformational changes. The kinetics analysis indicated at least two conformations, one of which induced by ATP binding to the high-affinity catalytic site, the other one induced by the ensuing ADP still bound at the same site after hydrolysis.

Based on the above results, the unisite catalysis can be hypothesized to constitute a “priming” reaction, which sets the stage for the subsequent rotary catalysis. Such a priming reaction at the high affinity site would generate either an ATP-bound or an ADP-bound conformation. Those two conformations may be taken to coincide with E_U_ and E_C_, respectively, since they are similarly induced by the ATP or ADP occupancy of the high-affinity site.

Cryo-EM studies in the presence or absence of multisite nucleotides (Sobti et al., 2019, 2020) showed that, after exposure to ATP, the *E. coli* enzyme adopts a different conformation, with a catalytic subunit (*β*_DP_) changing from open to closed, and the *ε*-subunit C-terminal domain (*ε*CTD) converting from the “up” to the “down” state. Conversely, exposure to ADP induced only a partial closure of the *β*_DP_ subunit, and maintained the *ε*CTD “up” state. Interestingly, in crosslinking studies, an “up” conformation of the bacterial enzyme was reported to retain synthesis but to be inhibited in hydrolysis, while the “down” conformation retained both (Tsunoda et al., 2001; Suzuki et al., 2003). The transition between the two forms was determined by the H^+^-gradient and by the ATP/ADP ratio (Suzuki et al., 2003). Though the *ε*CTD-less enzyme retained the nucleotide-dependent uncoupling (D’Alessandro et al., 2017), suggesting that the *ε*CTD is not the primary motor of the E_C_ ↔ E_U_ change, such two largely different *ε*CTD states imply two largely different ATP synthase conformations even in the *ε*CTD-less enzyme.

## 4 Leak, slip, or lower H^+^/ATP stoichiometry?

The lower H^+^/ATP coupling ratio of E_U_ could be due to a leak (a passive H^+^ channel), to more frequent slippage (a stochastic “deviation” from the usual coupled reaction pathway), or to a different coupling mechanism, featuring a lower H^+^/ATP stoichiometry—or to a combination of the three. Leak or higher slippage would mean that energy dissipation within E_U_ is significantly higher than energy dissipation within E_C_. Possibly, only the case of a lower H^+^/ATP stoichiometry, which does not necessarily involve a higher energy dissipation in E_U_, could succeed in explaining the mystery of ATP-Pi exchange (Section 2.3). A variable H^+^/ATP stoichiometry, on top of a slippage, has been reported in V-ATPases, which was able to explain the very steep H^+^-gradient found in the vacuoles of lemon fruit (Müller and Taiz, 2002). Future experimental work might confirm the V-ATPase result and eventually find similar evidence in the ATP synthase as well.

## 5 Conclusion

In the present work, some functional evidence is reviewed, indicating that the ATP synthase might adopt two different conformations, characterized by different H^+^/ATP coupling ratios, and that the ATP or ADP occupancy of its high-affinity catalytic site may be the key for the conformational switch (Section 2). In addition, structural evidence is reviewed, which supports the existence of two largely different conformations, according to the ATP or ADP occupancy of the high-affinity catalytic site (Section 3). Future work may confirm or not the idea that those two ATP synthase conformations differently couple nucleotide synthesis/hydrolysis with H^+^-translocation through F_O_. Should such modulation by the ATP/ADP ratio of the coupling degree between the two main energy currencies in the cell—ATP and the transmembrane H^+^-gradient—be confirmed, it would open yet another new perspective on the subtlety and elegance of both ATP synthase and of metabolism regulation.
